# T-cell mechanisms in atopic dermatitis 

**DOI:** 10.5414/ALX02630E

**Published:** 2026-06-03

**Authors:** Phila Cara Baumann, Lennart Matthias Roesner

**Affiliations:** 1 Department of Dermatology and Allergy, Hannover Medical School (MHH), and; 2 Cluster of Excellence RESIST (EXC 2155), Hannover Medical School, Hanover, Germany

**Keywords:** atopic dermatitis, atopic eczema, T cell, skin, inflammation, Th2, Th2a, resident memory T cell, skin-associated lymphoid tissue, autoallergy

## Abstract

This review provides an overview of recent advances in understanding T-cell inflammation in atopic dermatitis (AD), a common chronic inflammatory skin disease. After recognizing their cognate antigen in the acute phase of the disease, the homing of T cells from the circulation into the skin via cutaneous lymphocyte antigen (CLA) is the prerequisite to skin inflammation and subsequent systemic and local, tissue resident, memory (TRM) formation. Initial observations suggest that antigen presentation occurs in structures such as induced skin-associated lymphoid tissue (iSALT), in addition to lymphatic organs. Aside from environmental antigens, such as aeroallergens, other antigen sources also appear to play a role: humoral and cellular responses to microbial antigens and autoantigens are discussed to drive and shape skin inflammation in AD. In-depth characterization of differentiated, activated Th2 cells in AD shows their ability to recognize different signals from epithelial cells directly. The heterogeneity of patients with antigen sensitizations and T-cell phenotypes is believed to influence therapeutic success. This suggests that a more precise characterization of patient subgroups would enable targeted, individualized therapy.

## Atopic dermatitis 

Affecting up to 20% of children and ~ 3% of adults worldwide, varying among countries, atopic dermatitis (AD) is one of the most common chronic inflammatory skin conditions with relapsing flares [1]. Described as a multifactorial disease with a complex pathogenesis, its causes arise from genetic, immunological, and environmental factors and are associated with skin barrier and immune dysfunctions. Individuals with a family history of atopic diseases, especially AD, are at high risk, demonstrating the impact of genetic predisposition. Mutations in the filaggrin gene (FLG), which is crucial for maintaining a cutaneous barrier integrity, are a major genetic risk factor for AD [2]. AD often occurs alongside other atopic diseases, such as allergic asthma or allergic rhinoconjunctivitis. Exacerbation of AD can be triggered by various environmental stimuli, including allergens, microbial antigens, and superantigens. Stress, microbiome dysbiosis with increased *Staphylococcus aureus* colonization, and the itch-scratch cycle based on IgE-mediated histamine release can also contribute to AD aggravation [3]. Clinically, AD manifests as dry, itchy, and inflamed eczematous skin that relapses chronically, leading to a substantial impact on quality of life [2]. Immunologically, AD is characterized by increased activation and infiltration of immune cells, especially T cells, into the skin, followed by cytokine release. In addition, keratinocytes can release various cytokines that contribute to Th2 cell differentiation, leading to the maintenance of skin inflammation via a cytokine-mediated feedback loop between T cells and keratinocytes [4]. 

## Basic principles 

### Antigen presentation 

Antigens in the skin are recognized and ingested by Langerhans cells (LCs), immature dendritic cells (DCs) residing in the epidermis. The LCs then migrate to the peripheral lymph nodes where they differentiate into mature DCs with co-stimulatory activity able to prime naïve CD4^+^ and CD8^+^ T cells by presentation of the foreign antigen to the T cells via major histocompatibility complex (MHC) molecules [5]. Several factors determine the fate of the T cells, including cytokines, the activation of pattern recognition receptors (PRRs), and the microenvironment during antigen presentation. In Langerhans cells from AD skin, an increased signaling via the high-affinity IgE-receptor, PRRs, and lipid mediators takes place, supporting a Th2 polarization [6]. 

### T helper (Th) cells are part of the adaptive immune system and can be divided into distinct subtypes 

Th2 cells are a subset of CD4^+^ effector T cells producing mainly interleukin (IL)-4, IL-5, IL-9, IL-13, and IL-31 and are of importance for immune responses against parasites and for humoral immunity. AD is primarily considered a Th2-driven disease, as T cells of acute lesional skin secrete these interleukins, contributing to barrier dysfunction and pruritus, but also influencing IgE class switching and eosinophil recruitment [7, 8]. Two transcription factors that are crucial for regulating Th2 differentiation and production of type 2 cytokines are GATA binding protein 3 (GATA3) and signal transducer and activator of transcription 6 (STAT6). 

On the other hand, chronic lesions are dominated by Th1 cells, which under physiological conditions are required for immune protection against intracellular pathogens. Th1 cells promote cell-mediated immune responses and mainly secrete interferon-γ (IFN-γ), IL-2, and tumor necrosis factor-α (TNF-α). These cytokines can lead to the activation of macrophages and the elimination of microbial pathogens [7]. 

Furthermore, the role of Th17 and Th22 cells in the immunopathogenesis of AD has been described. Th22 cells secrete IL-22 and express the chemokine receptors CCR4 and CCR10, which are important for skin-homing and influence keratinocyte proliferation, thereby affecting the epidermis, contributing to hyperplasia and barrier dysfunction and resulting in a worsening of the skin during the chronic phase [9]. Th2/Th22 cells have been shown to be disease-specific for AD and are associated with severity scores [10], while Th17 cells are also present in AD skin and produce cytokines including IL-17A/F and IL-22 [11]. These cells have crucial functions in inflammation, neutrophil activation, and host defense, are more commonly present in Asian and African American patients, and may be associated with intrinsic AD as well as responses to certain allergens [7]. 

Additionally, regulatory T cells (Tregs) play an important role in suppressive immune responses but can differentiate into different Th subtypes, influenced by the predominant cytokine milieu. This may lead to a conversion from an immunosuppressive to a proinflammatory phenotype, finally contributing to AD pathogenesis [12]. 

CD8^+^ T cells have also been shown to play important roles in the initiation of skin flares [13], adaptive immune responses to allergens [14] and autoallergens [15] as well as further driving the disease by secreting cytokines including IL-13 [16, 17], which deserves special attention. 

### Skin homing of T cells and T_RM_ formation 

T cells are licensed to enter the skin from the circulation by expressing a set of surface molecules. The most prominent of these is the cutaneous lymphocyte-associated antigen (CLA), a carbohydrate modification of platelet selectin ligand-1 (PSGL-1), which has been shown to interact with E- and P-selectin on post-capillary venules in the skin. Similarly, lymphocyte function-associated antigen 1 (LFA-1) binding to ICAM-1 and integrin α4β1 interacting with VCAM-1 enable the extravasation into the skin. Furthermore, CLA^+^ T cells are attracted by chemokines recognized via CCR4 and CCR10 [18]. After resolution of a local inflammation, effector or central memory T cells that have recognized their cognate antigen are believed to either go into apoptosis or to build a local resident T-cell memory (T_RM_ cells). Providing local surveillance and defending against known pathogens, T_RM_ cells have gained attention through recent years. CD69 and CD103 are hallmarks of T_RM_, and function as retention factors in epithelial tissue. Additionally, skin T_RM_ cells usually express skin homing CLA [18]. Klicznik et al. [19] found evidence that blood CD4^+^CLA^+^CD103^+^ T cells had previously been skin-resident and had downregulated CD69 to re-enter circulation. 

## Cutting edge 

### Tertiary lymphoid structures in the skin? 

While the importance of the adaptive immune system for AD is undisputed, the question remains whether antigen presentation during the effector phase occurs solely in the primary and secondary lymphoid organs, such as the skin-draining lymph nodes, or if T_RM_ cells recognize their cognate antigens within the skin in tertiary lymphoid structures, following the concept of induced skin-associated lymphoid tissue (iSALT) by Ono and Kabashima [20, 21]. The observation of lymphoid-like structures within the skin by independent studies points to the latter: two-photon microscopy revealed DCs and T cells clustering in proximity, under the influence of perivascular macrophages [22] and high endothelial venules [23]. Furthermore, aggregates of T cells and LCs have been detected in atopy patch test-induced skin inflammation [24]. Nevertheless, direct evidence of antigen presentation to specific T cells within the skin has yet to be provided and will probably be difficult to obtain in humans. However, further investigation of this emerging concept is warranted. 

### Phenotyping subtypes of Th2 cells 

Th2 cells that express the key cytokines IL-4 and IL-13 as well as IL-5 are the primary drivers of the disease and the target of the biological dupilumab. Within the adaptive, TCR^+^ clonally expanded Th2 cells, a specific subtype has been identified as being particularly associated with allergic immune reactions. These Th2a-designated cells share expression of surface molecules characteristic for TCR-negative type 2 innate lymphoid cells (ILC2) and appear to possess the capacity to directly recognize signals from the epithelial cells via the ST2 (IL1RL1), thereby detecting the alarmin IL-33 [25] and subsequently secreting amphiregulin [26] (Table 1). 

**Figure 1:**
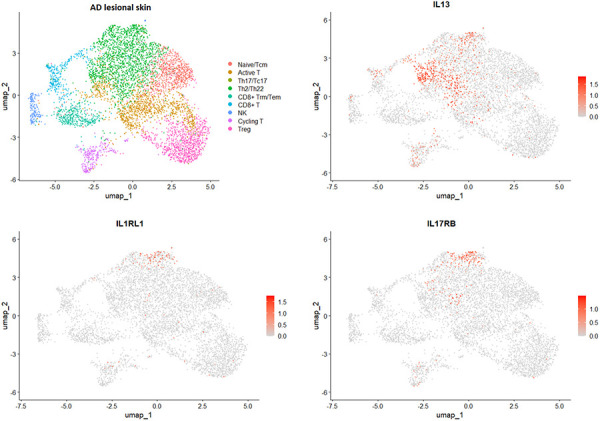
Atopic dermatitis (AD) lesional skin T cells harbor subgroups that express IL1RL1 (ST2) and IL17RB, which have been described as markers of Th2a cells. Single-cell RNA sequencing data of T cells isolated from lesional skin of 10 patients with AD; dataset from [10].

Signaling through CRTh2, a G-protein–coupled receptor of prostaglandin D2 (PGD_2_), additionally leads to the secretion of Th2 cytokines. Consequently, Th2a cells combine features of adaptive α/β T cells and direct quick responsiveness of ILC2. Expression of the costimulatory molecule KLRB1 (CD161), a further characteristic of ILC2 and Th2a, allows specific modulation of these cells. Th2a cells express only low amounts of CD27, showing their differentiation into the effector state, but express detectable levels of the receptors TSLPR and IL17RB and are thereby capable of sensing the epithelial proinflammatory cytokines TSLP and IL-25 [25, 27]. Recently it was observed that Th2a cells are able to sense IL-18 via its receptor IL18Ra, whose expression is upregulated by IL-9, a pathway that further enhances the secretion of IL-13 [28]. Due to a clonal overlap to Th2 cells, it was hypothesized that Th2a represents a highly polarized and terminally differentiated type of effector cell [29]. These observations, which have mainly been made in patients with respiratory allergies, appear to be transferrable to AD, since expression of the aforementioned markers can be detected in AD lesional skin T cells as well (Figure 1). 

Th2a cells have gained further attention since they have been found to decline specifically during successful allergen immunotherapy [30], but to reside within the skin during dupilumab therapy [31]. To investigate whether these cells may cause symptoms to reappear after therapy discontinuation, or whether they are biomarkers of treatment resistance, longitudinal studies with paired tissue and blood samples are needed. 

### Autoallergy 

Autoallergy is being discussed as a key mechanism in the complex pathogenesis of AD, contributing to its chronicity and the atopic march [32]. It refers to cellular and humoral type 2 immune responses to the body’s own proteins, which have therefore been termed autoallergens. It is believed that tissue damage derived from mechanical scratching, infections, and chronic inflammation can expose self-epitopes that are otherwise hidden from the immune system and that are then taken up, processed, and presented by antigen-presenting cells. Other autoantigens may also be presented under physiological conditions [33] but evoke a pro-inflammatory response only after a sensitization process, which can occur due to cross-reactivity to environmental allergens. For example, T cells specific the fungal thioredoxin (Mala s 13) of the skin-colonizing yeast *Malassezia sympodialis* and its homologous human thioredoxin (hTrx) have been shown to be cross-reactive in sensitized AD patients. In addition, fungal and human manganese superoxide dismutases exhibit strong structural similarity and cross-reactivity [33]. The occurrence of autoallergen-specific IgE autoantibodies correlates with the severity of AD, presence of atopic comorbidities, and environmental factors [32]. Nevertheless, further exploration is needed to determine the potential value of IgE autoantibodies as predictive biomarkers for the course of AD. 

### T-cell response to the skin microbiome 

During recent years, decent insights into the T-cell response to the skin microbiome have been gained. A non-invasive murine model of AD could elegantly show the potential of *S. aureus* to induce T_RM_ formation in the skin, delivering protection from infection but also exacerbating inflammatory skin disease [34]. In patients, the adaptive immune response to secreted staphylococcal factors shows, interestingly, a phenotype that is on the fringe of a proper pathogen response and a type 2 allergic phenotype [35, 36]. This type-2 immune response induced by bacteria can be seen as an immune evasion strategy, matching to the observation that *S. aureus* influences the development of macrophages towards a phenotype with reduced capabilities to fight this pathogen [37]. Aside from epitope presentation via MHC, also lipid antigens can be presented to T cells via CD1a, and a specific expansion of CD1a-restricted T cells in response to *S. aureus* was observed in AD patients [38]. 

## Relevance for the clinician/aspects for clinical practice 

The approval of dupilumab as the first biologic drug for the treatment of moderate to severe AD in 2017 marked a new era in therapy of the disease. The humanized antibody became the first drug to specifically inhibit the central inflammatory axis (IL-4/IL-13) of AD, more precise by preventing the cytokines IL-4 and IL-13 from binding to the IL-4R α chain. The downstream signaling via the JAK/STAT6 pathway is thereby diminished, resembling a targeted interruption of the type 2 immune response. Three further therapeutic antibodies that target Th2-cell cytokines have been approved for AD to date. Tralokinumab and lebrikizumab target IL-13, and nemolizumab targets IL-31 via its receptor. In addition to the four biologics, conventional immunosuppressants such as cyclosporin A or methotrexate, and three Janus kinase inhibitors (JAKis), are available for the treatment of AD. Since JAKis do not specifically target the type 2 immune response, but also block signaling by other receptors, the anti-inflammatory mode of action is considered to be broader, thereby also affecting immunity against pathogens [39]. During recent years, several immune mediators have been investigated as therapeutical targets in clinical trials, e.g. TSLP, IL-33, IL-17, IL-22, IL-22Ra, IL-31R, IL-12/IL-23p40, H4R, IL-2, OX40, and OX40L [40]. Like JAKis, the latter two are believed to act on several T-cell subtypes and have shown particularly promising results. The induction of Tregs via IL-2 also appears promising [41]. Given the heterogeneity of T-cell phenotypes in AD patients, with also Th1, Th17, and Th22 cells playing important roles in at least subgroups, patients would benefit from personalized medicine targeting the respective pathogenic T-cell population. 

## Authors’ contributions 

The article was written jointly by PCB and LMR, LMR performed analyses and generated Figure 1. 

## Funding 

None. 

## Conflict of interest 

LMR has received institutional research grants from Almirall and Novartis and has participated in advisory boards for Almirall and Bencard. 

**Table 1.** Comparison of Th2a, conventional Th2, and type-2 innate lymphoid cells.

	**Th2a**	**Conventional Th2**	**ILC2**
T-cell receptor	Yes	Yes	No
Clonal propagation	++	+	–
Relevant surface markers	CD3^+^, CD4^+^, CCR4^+^, CCR6^−^, CXCR3^−^, CRTH2^+^, IL-17RB^+^, ST2^+^, IL-4Rα^+^	CD3^+^, CD4^+^, CCR4^+^, CCR6^-^, CXCR3^−^, CRTH2^+^ (variable), IL-4Rα^+^	CD127^+^, CRTH2^+^, ST2^+^, IL-4Rα^+^
Key trigger factors	TCR engagement, IL-4, epithelial alarmins (IL-33, IL-25, TSLP), PGD_2_	TCR engagement, IL-4	IL-33, IL-25, TSLP, IL-4, PGD_2_
Dominant effector cytokines/factors	IL-13, IL-9, IL-5, IL-4, amphiregulin	IL-13, IL-5, IL-4, IL-9 (variable)	IL-13, IL-5, IL-9, IL-4 (moderately), amphiregulin
Role in AD pathogenesis	Pathological type 2 immune responses to antigens, influenced by epithelial damage	Pathological type 2 immune responses to antigens	Early-phase amplification of type 2 inflammation; bridges epithelial damage to adaptive immunity
